# Added value of CRP to clinical features when assessing appendicitis in children

**DOI:** 10.1080/13814788.2022.2067142

**Published:** 2022-05-10

**Authors:** Guus C. G. H. Blok, Eelke D. Nikkels, Johan van der Lei, Marjolein Y. Berger, Gea A. Holtman

**Affiliations:** a Department of General Practice and Elderly Care Medicine, University Medical Center Groningen, University of Groningen, Groningen, The Netherlands; b Department of Medical Informatics, Erasmus Medical Center, Rotterdam, The Netherlands

**Keywords:** Primary health care, C-reactive protein, appendicitis, child, abdominal pain, point-of-care testing

## Abstract

**Background:**

The diagnostic value of C-reactive protein (CRP) for appendicitis in children has not been evaluated in primary care. As biochemical responses and differential diagnoses vary with age, separate evaluation in children and adults is needed.

**Objectives:**

To determine whether adding CRP to symptoms and signs improves the diagnosis of appendicitis in children with acute abdominal pain in primary care.

**Methods:**

A retrospective cohort study in Dutch general practice. Data was collected from the Integrated Primary Care Information database between 2010 and 2016. We included children aged 4–18 years, with no history of appendicitis, presenting with acute abdominal pain, and having a CRP test. Initial CRP levels were related to the specialist’s diagnosis of appendicitis, and the test’s characteristics were calculated for multiple cut-offs. The value of adding CRP to signs and symptoms was analysed by logistic regression.

**Results:**

We identified 1076 eligible children, among whom 203 were referred for specialist evaluation and 70 had appendicitis. The sensitivity and specificity of a CRP cut-off ≥10 mg/L were 0.87 (95%CI, 0.77–0.94) and 0.77 (95%CI, 0.74–0.79), respectively. When symptoms lasted > 48 h, this sensitivity increased to 1.00. Positive predictive values for CRP alone were low (0.18–0.38) for all cut-off values (6–100 mg/L). Adding CRP increased the area under the curve from 0.82 (95%CI, 0.78–0.87) to 0.88 (95%CI, 0.84–0.91), and decision curve analysis confirmed that its addition provided the highest net benefit.

**Conclusion:**

CRP adds value to history and physical examination when diagnosing appendicitis in children presenting acute abdominal pain in primary care. Appendicitis is least likely if the CRP value is < 10 mg/L and symptoms have been present for > 48 h.

KEY MESSAGES

- Testing CRP in children presenting with acute abdominal pain in primary care adds value to clinical features of appendicitis.
- Appendicitis is unlikely if the CRP value is < 10 mg/L and symptom duration is > 48 h.
- When suspicion of appendicitis is low, a CRP test is not clinically relevant.

## Introduction

Acute abdominal pain is a common symptom reported in 9% of consultations with children in primary care [1]. Although appendicitis is rare in these children (<5%) and may even resolve spontaneously, it can progress to perforation and death if undiagnosed [2–4]. It remains a diagnostic challenge for general practitioners (GPs) to differentiate appendicitis from common self-limiting or functional abdominal conditions that present similarly [5]. This is compounded by the difficult trade-off between trying to avoid unnecessary investigation and referral for abdominal surgery and not missing a case of appendicitis. Thus, a simple and readily available test could help GPs reduce doubt.

C-reactive protein (CRP) levels increase rapidly during acute inflammation [6]. In specialist care, CRP is of moderate diagnostic value for appendicitis, having a sensitivity of 0.62–0.85 and a specificity of 0.59–0.94 at *a* ≥ 10 mg/L cut-off value [7–10]. As a readily available point-of-care test (POCT), CRP is often used by GPs, including for children with acute abdominal pain [11]. However, the diagnostic accuracy of CRP has not been determined in primary care and we cannot generalise from the results for specialist care because of differences in patient spectrum, i.e. disease prevalence, severity, and distribution [2,12].

We aimed to determine the diagnostic characteristics of CRP testing for appendicitis in primary care and to assess the value of adding CRP to basic clinical assessment.

## Methods

### Design

In this retrospective cohort study, we included children with acute abdominal pain who underwent CRP tests ordered by a GP between November 2010 and November 2016. Data was sourced from the Dutch Integrated Primary Care Information (IPCI) database, which contains pseudonymised medical records for 1.5 million patients from 600 practices across the Netherland and has been used extensively for research [13]. We used data from three of the six software platforms within the IPCI database for this study. Data from the other three software platforms had already been used in another research project about the management of acute abdominal pain because these contained extra secondary care information, which was not evaluated in the present study [14].

### Study population

The International Classification for Primary Care (ICPC) is used for diagnostic coding in Dutch primary care. We manually reviewed the first patient contact in the study period that met all of the following criteria at the time of contact: the patient received a gastrointestinal diagnosis (ICPC codes D01 through D99); abdominal pain was mentioned in the free text record; the patient had been registered in that practice for at least 12 months; the patient was aged 4–18 years; and the GP obtained a CRP. We subsequently reviewed the identified contacts and retained only patients presenting with recent acute abdominal pain (i.e. the presenting symptoms started ≤1 week before the consultation). Patients with a history of appendicitis or appendectomy were removed.

### CRP test and clinical features

CRP levels were extracted automatically from laboratory results or manually from free-text entries. Data for age, gender, and body temperature were extracted automatically, while data for another 18 clinical features (symptoms and signs) described in seven clinical prediction rules were extracted by manual review [15]. Nausea and vomiting were combined into one variable consistent with most prediction rules. Elevated temperature and temperature ≥ 37.3 °C were combined into one variable according to the Alvarado score [16]. Based on a Dutch guideline, rebound tenderness, guarding, rigidity, and pain at jarring motions were combined as ‘peritoneal irritation’ [2,8]. Coders determined whether each clinical feature was present, absent, or not recorded (Supplementary Table 1). If in doubt, the coders discussed with an experienced GP (CGHB) or within the research team when doubt remained until consensus was reached.

### Appendicitis

The outcome of interest was appendicitis diagnosed by a medical specialist within six weeks after the initial consultation. The absence of appendicitis was based on either the secondary care specialist report or the GPs medical records during this period. When coders were in doubt, an expert panel of two experienced GP’s (MYB, CGHB) verified the diagnosis based on free text records and letters from the medical specialist.

### Statistical analysis

#### Diagnostic test characteristics of CRP

We calculated the following diagnostic characteristics for CRP testing with their 95% confidence intervals (95%CIs): sensitivity, specificity, positive likelihood ratio, negative likelihood ratio, positive predictive value, and negative predictive value. We determined that a sample of 1397 children with acute abdominal pain was needed to include 61 children with appendicitis and allow us to calculate sensitivity with sufficient precision (half-width of the 95%CI is 0.1). This was based on the reported 4.4% prevalence of appendicitis in primary care^2^ and an expected sensitivity of 0.80 using the typical 10 mg/L cut-off [7,17]. Subgroup analyses were performed by gender, age (4–8, 9–12, and 13–18 years), and symptom duration (<24, 24–28, and >48 h) [18]. We also calculated the test characteristics for CRP cut-off levels of ≥6, ≥20, ≥30, ≥40, ≥50, ≥80 and ≥100 mg/L. However, we did not consider a cut-off level of ≥5 mg to be informative because POCT values <5 mg/L are recorded as 5 mg/L in the Dutch Integrated Primary Care Information database.

#### Added value above symptoms and signs

We analysed the value of adding CRP to clinical features by comparing a logistic regression model that contained clinical features only (basic model) with one that added CRP to the basic model. The dependent variable in both models was appendicitis. Predictors recorded in > 50% of children were entered into the basic model without further selection as all predictors were based on literature and clinical practice [19,20]. To evaluate the performance of both models and hence the added value of CRP, we compared the areas under the curves (AUCs) and used decision curve analysis. Net benefits were calculated for a range of thresholds, with an upper limit of 0.40 [21].

#### Missing values

There was no missing demographic, CRP, or diagnostic data but there were missing values for some clinical predictors. We assessed the missing data mechanisms and patterns to exclude missing not at random [22], and if excluded, used multiple imputation (predictive mean matching, 10 iterations) with all clinical features, referrals, and outcomes to predict the missing data and construct 20 data sets. Rubin’s rule was used to calculate pooled AUCs with 95%Cis [23], and DeLong’s method was used to test the difference between models in each imputed dataset [23]. If the decision curves were similar for the 20 imputed datasets, we presented one randomly selected figure. A sensitivity analysis was performed by comparing the AUC of both models with complete case analysis only and a zero imputation analysis dataset in which missing values were replaced with zero (i.e. the assumption that the missing clinical predictor was absent). STATA/SE 16.1 (Stata Corp, USA) was used to calculate CRP-test characteristics, compare AUCs and construct decision curves. IBM SPSS version 26.0 (IBM Corp, Armonk, NY, USA) was used for all other analyses.

## Results

### Study population and diagnosis

We identified 2741 children for manual review. Among these, 1076 had presented for the first time with acute abdominal pain and had CRP levels measured, with 70 (6.5%) having appendicitis (13 had a perforated appendix). The prevalence of appendicitis was 10.2% (95%CI, 7.8%–13.4%) in boys and 3.7% (95%CI, 2.5%–5.5%) in girls (Table 1). Other emergencies were detected, including one case each of hydronephrosis, intussusception, abdominal lymphoma, and pneumonia. In total, 265 of the 1076 children (25%) had no missing predictors, and except for having a greater prevalence of appendicitis, these were comparable to patients with missing values (Supplementary Table 2).

**Table 1.** Patient characteristics clinical features and CRP by diagnosis.

Characteristics	Total study population (*N* = 1076)	No appendicitis (*N* = 1006)	Appendicitis (*N* = 70)
Male, *n*/total *N* (%)	459/1076 (42.7%)	412/1006 (41.0%)	47/70 (67.1%)
Age in years, median (IQR)	13.00 (IQR 6)	13.00 (IQR 4)	13.00 (IQR 6)
4–8 years, *N*/total *N* (%)	190/1076 (17.7%)	184/1006 (18.3%)	6/70 (8.6%)
9–12 years, *N*/total *N* (%)	303/1076 (28.2%)	281/1006 (27.9%)	22/70 (31.4%)
13–18 years, *N*/total *N* (%)	583/1067 (54.2%)	541/1006 (53.8%)	42/70 (60.0%)
Clinical features, *N*/total *N* (%)			
Symptoms			
Pain duration			
<24 h	229/823 (27.8%)	211/762 (27.7%)	18/61 (29.5%)
24–48 h	78/823 (9.5%)	67/762 (8.8%)	11/61 (18.0%)
48 h	516/823 (62.7%)	484/762 (63.5%)	32/61 (52.5%)
Nausea/vomiting	384/575 (66.8%)	338/521 (64.9%)	46/54 (85.2%)
* Signs*			
Reduced bowel sounds	70/578 (12.1%)	61/536(11.4%)	9/42 (21.4%)
Peritoneal irritation	193/725 (26.6%)	153/664 (23.0%)	40/61 (65.6%)
Tenderness RLQ	415/699 (59.4%%)	372/646 (57.6%)	43/53 (81.1%)
Elevated temperature	227/718 (31.6%)	193/662 (29.2%)	34/56 (60.7%)
Investigations, median (IQR)			
CRP in mg/L (*N* = 1076)	5.00 (IQR: 2–12)	5.00 (IQR: 2–8)	42.00 (IQR: 18–83)

The numerator is the number of children in whom the feature is present; the denominator is the number of children in whom the feature is recorded. CRP: C-reactive protein; IQR: interquartile range.

### CRP

The median CRP value was higher in children with than without appendicitis at 42 mg/L (IQR: 18–83) and 5 (IQR:2 − 8 mg/L, respectively (Table 1). All children with a perforation had CRP levels > 20 mg/L (median 96 mg/L). At a cut-off of ≥ 10 mg/L, the sensitivity for appendicitis was 0.87 (95%CI, 0.77–0.94) and the specificity was 0.77 (95%CI, 0.74–0.79). The positive predictive value for CRP alone ranged from 0.18 (95%CI, 0.14–0.22) to 0.38 (95%CI, 0.22–0.56) at the minimum and maximum cut-off values of 6 mg/L and 100 mg/L, respectively (Table 2). When symptoms had lasted < 24 h, the sensitivity of CRP was only 0.67 (95%CI: 0.41–0.87) but when they had lasted > 48 h, it increased to 1.00 (95%CI, 0.89–1.00); by contrast, the specificity remained stable at 0.77 (0.71–0.82) and 0.75 (0.71–0.79), respectively. The diagnostic test characteristics were similar between gender and age groups (Supplementary Table 3).

**Table 2.** Test characteristics of CRP for appendicitis in children with acute abdominal pain at different cut-off levels.

Cut-off	Sensitivity (95%CI)	Specificity (95%CI)	LH+ (95%CI)	LH− (95%CI)	PPV (95%CI)	NPV (95%CI)
≥6 mg/L	0.90 (0.81–0.96)	0.71 (0.68–0.73)	3.05 (2.69–3.45)	0.14 (0.07–0.29)	0.18 (0.14–0.22)	0.99 (0.98–1.00)
≥10 mg/L	0.87 (0.77–0.94)	0.77 (0.74–0.79)	3.76 (3.26–4.35)	0.17 (0.09–0.31)	0.21 (0.16–0.26)	0.99 (0.98–1.00)
≥20 mg/L	0.74 (0.62–0.84)	0.85 (0.82–0.87)	4.85 (3.97–5.93)	0.30 (0.20–0.45)	0.25 (0.20–0.32)	0.98 (0.97–0.99)
≥30 mg/L	0.63 (0.51–0.74)	0.89 (0.87–0.91)	5.65 (4.39–7.26)	0.42 (0.31–0.57)	0.28 (0.21–0.36)	0.97 (0.96–0.98)
≥40 mg/L	0.54 (0.42–0.66)	0.92 (0.90–0.94)	6.91 (5.11–9.35)	0.50 (0.38–0.61)	0.33 (0.24–0.42)	0.97 (0.95–0.98)
≥50 mg/L	0.41 (0.30–0.54)	0.94 (0.93–0.96)	7.19 (4.94–10.4)	0.62 (0.51–0.76)	0.33 (0.24–0.44)	0.96 (0.94–0.97)
≥80 mg/L	0.27 (0.17–0.39)	0.97 (0.95–0.98)	8.03 (4.84–13.3)	0.75 (0.65–0.87)	0.36 (0.23–0.50)	0.95 (0.94–0.96)
≥100 mg/L	0.19 (0.11–0.30)	0.98 (0.97–0.99)	8.90 (4.65–17.0)	0.83 (0.74–0.93)	0.38 (0.22–0.56)	0.95 (0.93–0.96)

CI: Confidence interval; CRP: C-reactive protein; LH+: positive likelihood ratio; LH−: negative likelihood ratio; NPV: negative predictive value; PPV: positive predictive value.

### Value of adding CRP to clinical features

**Figure 1.**
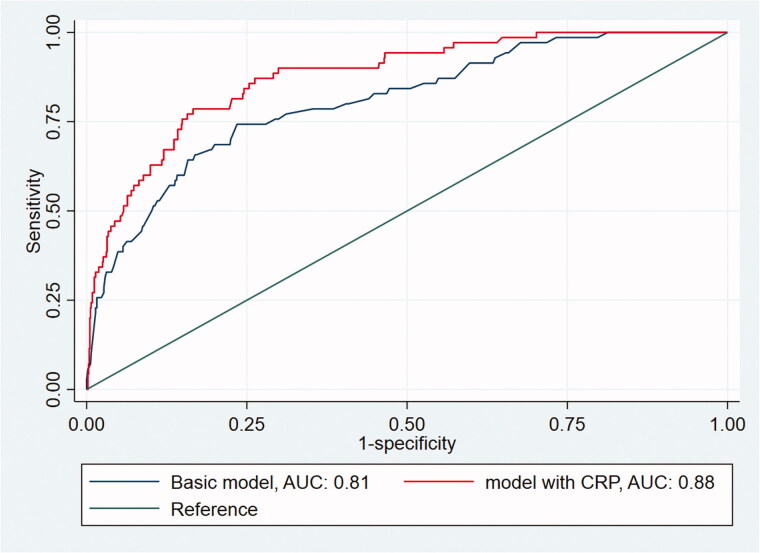
AUC curve of randomly chosen datasat # 14, comparing the AUC of the basic model and the model with CRP.

**Figure 2.**
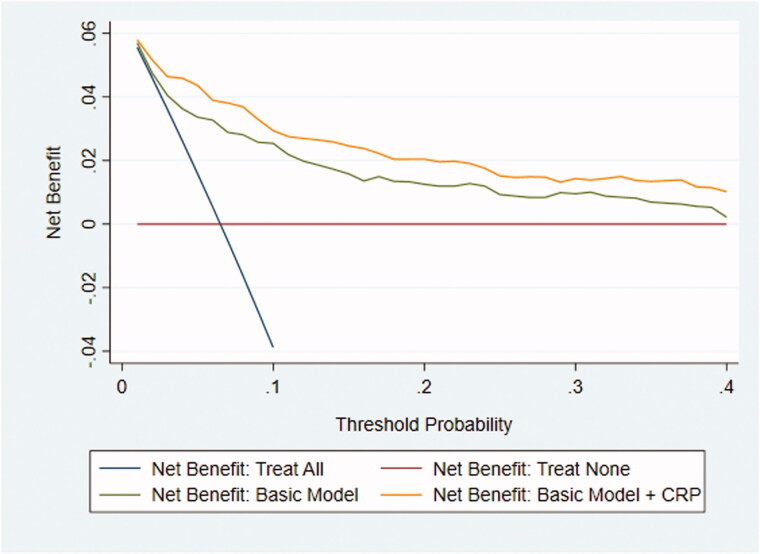
Decision curve showing the net benefit of referral based on the basic prediction model with and without CRP (imputed dataset #14). In 15 of the 20 imputed datasets the basic model with the addition of CRP had greater net benefit for all threshold probabilities compared with the basic model alone.

The following predictors were recorded in > 50% of the participating children and were included in the basic model: pain duration (76%), elevated temperature (68%), peritoneal irritation (67%), right lower quadrant tenderness (65%), bowel sounds (54%), and nausea/vomiting (53%) (Supplementary Table 4). When all predictors of the basic model were negative, the predicted risk of appendicitis was 0.002; adding CRP in this context increased the risk of appendicitis to 0.05 for a CRP value of 100 mg/L. Notably, adding CRP to the basic model increased the AUC significantly from 0.82 (95%CI, 0.78–0.87) to 0.88 (95%CI, 0.84–0.91) (Figure 1). In the sensitivity analyses, the AUC still increased significantly from the basic model to the basic plus CRP model, as follows: from 0.82 (95%CI, 0.72–0.91) to 0.89 (95%CI, 0.82–0.97) using complete case analysis (*n* = 219) and from 0.84 (95%CI, 0.80–0.89) to 0.89 (95%CI, 0.86–0.93) using zero imputation. The decision curves indicated that the net benefit of the model with CRP added was higher than that for the basic model alone at each referral threshold (Figure 2).

## Discussion

### Main findings

When differentiating appendicitis in children presenting with acute abdominal pain in primary care, a CRP cut-off at ≥ 10 mg/L had a sensitivity and specificity of 0.87 and 0.77, respectively. However, the sensitivity increased to 1.00 when symptoms had been present for > 48 h, with a negative test making appendicitis less likely. It was notable that all children with perforation had a CRP > 20 mg/L but the utility of this finding will need further investigation. Adding CRP to the basic clinical model increased the AUC from 0.82 to 0.88, with decision curves confirming the added benefit.

### Comparison with existing literature

We found no other study looking at the diagnostic value of CRP for appendicitis in children with abdominal pain in primary care. Interestingly, registration data could be used because enough CRP tests were performed despite not being recommended in the Dutch guideline [2]. At the ≥ 10 mg/L cut-off, although the sensitivity was higher than reported in specialist care (0.87 vs 0.62–0.85), the specificity was comparable (0.77 vs 0.59–0.94) [7–10]. Sensitivity was also higher among children with symptoms for > 48 h, consistent with other research showing that the sensitivity and discrimination of CRP increased over the first few days [24]. Consequently, a low CRP value should be interpreted with caution if symptoms have only developed recently.

The decision curve confirmed that adding a CRP test was beneficial across a range of clinically reasonable referral thresholds. Adding a CRP test may therefore improve decision-making for GPs who adopt both high (to avoid negative referrals) and lower (to avoid missing appendicitis) referral thresholds [21]. Although no previous study has separately evaluated the value of adding CRP to other appendicitis features, we note that it was selected for use as a predictor in the Appendicitis Inflammatory Response score in secondary care based on logistic regression analysis [25]. In that setting, CRP is tested routinely in cases of suspected appendicitis, with imaging recommended before deciding to perform appendectomy [26]. The present study adds to the existing literature, demonstrating a clear benefit from adding CRP to signs and symptoms when predicting appendicitis in children in primary care.

### Strengths and limitations

This study benefitted from including enough patients with appendicitis to calculate sensitivity with sufficient precision. A prospective cohort study would not have been feasible due to the low prevalence. Although we included fewer patients than required by our sample size calculation, the prevalence of appendicitis was higher than expected [2], possibly because GPs used the CRP test when they had a higher suspicion of appendicitis. Nevertheless, these results are only applicable to children with acute abdominal pain in whom the GP considers ordering a CRP test. We also used ≥ 10 mg/L as the main cut-off level despite there being no consensus on the optimal cut-off in acutely ill children [27]. Although adding CRP to the model resulted in a statistically significant increase in the AUC, this does not necessarily imply clinical relevance. Therefore, decision curve analysis enabled us to quantify the clinical benefit of adding CRP to the model [28].

Using routine healthcare data introduced essential limitations [20]. First, the clinicians who coded the final diagnosis were not blinded to the CRP values, potentially leading to overestimating information bias and diagnostic accuracy. We used specialist reports to ascertain the final diagnosis, when available but had to rely on free text entries in some cases. Second, one predictor had 47% of its values missing, which we handled by multiple imputations. However, sensitivity analyses using zero imputation and complete cases produced similar improvements in the AUCs after adding CRP. Third, because we evaluated routine practice data, not all patients will have received the same reference standard, with diagnosis verified by operation, imaging, or observation in different cases. This may introduce differential verification bias or workup bias that affected the test characteristics if mild or spontaneously resolving cases of appendicitis were misclassified [29]. However, given that these children do not need an operation, missing them has limited clinical impact. Fourth, it can be challenging to select the proper patient population retrospectively. As the database contained problem-oriented records, we were able to select children with acute abdominal pain. However, we only selected children with a CRP test which implies that the GP was unsure about the diagnosis and that a CRP-test was available. Although generalising the results to all children with acute abdominal pain would introduce selection bias, the results can be generalised to children in which the GP is in doubt whether or not to refer. In a previous cohort study, children with appendicitis were less likely to be tested for CRP than children with appendicitis [14]. Furthermore, as all consultations took place from 2010 to 2016, CRP-testing may have become more available to the GP. However, the Dutch guideline still advises against CRP-testing for children with suspected appendicitis. Finally, we did not analyse the diagnostic value of CRP for outcomes other than appendicitis, so our conclusions are limited to appendicitis. However, the task of the GP is not to diagnose acute appendicitis but to differentiate between severe and not threatening symptoms and signs on presentation in primary care. Since only four children with another diagnosis that needed emergency care were present, analysis for the outcome emergency (including appendicitis) would have yielded similar results.

### Implications for research and practice

CRP adds value to symptoms and signs alone and may improve decision-making by GPs, but it should only be ordered when indicated by clinical history and physical examination. Indeed, a CRP test in children with acute abdominal pain but no signs or symptoms of appendicitis is meaningless. The GP can use either a POCT or an external laboratory, with both yielding similar results [30], though POCT is available more rapidly and can reduce the risk of perforation.

None of the cut-offs had optimal sensitivity or specificity for safely excluding appendicitis. Even at ≥ 10 mg/L, 13% of cases had missed appendicitis (though perforation was less likely). However, conservative management may be supported at this threshold, especially if symptoms have lasted > 48 h. If the child is sent home on this basis, GPs should offer clear safety-netting advice about the diagnostic uncertainty, alarm symptoms, and need to reassess. At a cut-off level of ≥ 100 mg/L, only 2% of children without appendicitis tested positive, indicating that at this level, or possibly lower, referral is highly indicated. It should be noted that the confidence intervals around the sensitivity were relatively wide, which means that there is uncertainty about how often acute appendicitis will be missed. However, the number of negative referrals is determined by the false positive rate (1-specificity), which was estimated with high precision.

Given that CRP added value to routine assessment, we must now consider how to include it in a clinical prediction rule. It is also unknown if testing with or without a clinical prediction rule affects GPs decisions and patient outcomes. Therefore, before recommending CRP in primary care, the impact of its use on patient outcomes should be evaluated in a randomised controlled trial.

## Conclusion

In conclusion, adding CRP to symptoms and signs may help GPs decide whether to refer a child with suspected appendicitis to secondary care. Studies are needed to evaluate whether this test can improve decision-making in children with acute abdominal pain.

## Supplementary Material

Supplementary Table 1

Supplementary material: Decision curve analysis

Supplementary Table 4

Supplementary Table 3

Supplementary Table 2
