# Correction

**DOI:** 10.1080/13814788.2022.2155364

**Published:** 2022-12-12

**Article title:** Added value of CRP to clinical features when assessing appendicitis in children

**Authors:** Blok, C. G. H., Nikkels, E. D., van der Lei, J., Berger, M. Y., & Holtman, G. A.

**Journal:** *European Journal of General Practice*

**Bibliometrics:** Volume 28, Number 1, pages 95–101

**DOI:** https://doi.org/10.1080/13814788.2022.2067142

When this article was first published online there was an error in the following sentence in the ‘Value of adding CRP to clinical features’ section (p. 98):

When all predictors of the basic model were negative, the predicted risk of appendicitis was 0.002; adding CRP in this context increased the risk of appendicitis to 0.05 for a CRP value of 100mg/L.

This sentence has been corrected to:

When all predictors of the basic model were negative, the predicted risk of appendicitis was 0.002; adding CRP in this context increased the risk of appendicitis to 0.013 for a CRP value of 100mg/L.

