# Supplementary material for: Added value of CRP to clinical features when assessing appendicitis in children
Source: Eur J Gen Pract. 2022 May 10;28(1):95–101. doi: 10.1080/13814788.2022.2067142 (PMC9103685; doi:10.1080/13814788.2022.2067142)
Supplement: Supplementary Table 1 [file IGEN_A_2067142_SM6362.docx]

**Supplementary Table 1**. Summary of scoring criteria for features of appendicitis

| **Symptoms** | **Present** | **Absent** | **Not recorded** |
| --- | --- | --- | --- |
| **Pain in RLQ** | Pain localised in RLQ  Abdominal pain including RLQ | Pain localisation other than RLQ | No record of pain RLQ |
| **Pain migration to RLQ** | Pain RLQ, combined with description of migration | Constant localisation of pain | No record of migrating pain |
| **Pain intensity** | Severe pain: VAS 7–10 | Mild pain: VAS 1–6 | Pain severity not stated |
| **Pain duration <24 hours** | Pain shorter than 24 hours or 1 day | Pain 24 hours (one day) or longer | Duration of pain not stated |
| **Increasing pain** | Increasing pain | Constant or decreasing pain | No record of change in pain intensity |
| **Nausea/Vomiting**  **(combined variable)** | Nausea or vomiting present | Nausea or vomiting absent (none present) | Nausea nor vomiting recorded |
| **Anorexia** | Decreased food intake or ketones in urine | Normal food intake and no presence of ketones in urine | Food intake or ketones in urine not recorded |
| **Diarrhoea** | Diarrhoea | Absence of diarrhoea | Diarrhoea not recorded |
| **Constipation** | Constipation or abdominal mass found | Absence of constipation and no abdominal mass found | Constipation not recorded and abdominal mass not recorded |
| **Signs** |  |  |  |
| **RLQ tenderness** | Tenderness of RLQ or abdominal tenderness including RLQ | Absence of tenderness or tenderness other than RLQ | Tenderness not recorded |
| **Abnormal bowel sounds** | Abnormal or absent bowel sounds | Normal bowel sounds | Bowel sounds not recorded |
| **Elevated temperature** | Elevated temperature or fever  Temperature 37.3°C or higher measured | Absence of elevated temperature or fever  Temperature lower than 37.3°C measured | Temperature or fever not recorded  No temperature measurement |
| **Difficulty walking** | Difficulty walking | Normal walking | Difficulty walking not recorded |
| **Peritoneal irritation**  **(combined variable)** | Rebound tenderness or Guarding or Rigidity or Pain at jarring motions present | Rebound tenderness or Guarding or Rigidity or Pain at jarring motions absent (and none of these present) | Rebound tenderness nor Guarding nor Rigidity nor Pain at jarring motions recorded |

Abbreviations: RLQ, right lower quadrant; VAS, visual analogue score

Source: C G H Blok et al., Appendicitis in children with acute abdominal pain in primary care, a retrospective cohort study, *Family Practice*, 2021, 38 (6), 758–765, by permission of Oxford University Press.
