# Supplementary material for: Added value of CRP to clinical features when assessing appendicitis in children
Source: Eur J Gen Pract. 2022 May 10;28(1):95–101. doi: 10.1080/13814788.2022.2067142 (PMC9103685; doi:10.1080/13814788.2022.2067142)
Supplement: Supplementary material: Decision curve analysis [file IGEN_A_2067142_SM8594.docx]

Decision curve analysis is a method to compare the clinical benefit of different predictive models. It is based on the idea that clinicians assign different value to true positive and false positive referrals. For instance, a GP may be prepared to refer maximally five children to find one case of acute appendicitis. In this case, the referral threshold lies at a probability of 0.2 and four negative referrals balance out one referral for acute appendicitis. When the predicted probability of appendicitis is higher than the referral threshold, the expected net benefit is greater than zero and the clinician will refer the child. In decision curve analysis the net benefit increases by 0.01 when the model identifies one extra true positive case in 100 children in the cohort. Conversely, net benefit decreases when the model identifies extra false positive cases, depending on the referral threshold. In the decision curve the net benefit of different models is plotted for a range of thresholds. The better model has a higher net benefit at the referral threshold that the clinician wants to use or, when this threshold is unknown, over a range of thresholds.
