# Supplementary material for: Added value of CRP to clinical features when assessing appendicitis in children
Source: Eur J Gen Pract. 2022 May 10;28(1):95–101. doi: 10.1080/13814788.2022.2067142 (PMC9103685; doi:10.1080/13814788.2022.2067142)
Supplement: Supplementary Table 4 [file IGEN_A_2067142_SM8593.docx]

**Supplementary Table 4.** Analysis of the basic model with and without CRP and multiple diagnostic odds ratios of predictors.

| **Predictors** | **Basic model**  **OR (95%CI)** | **Basic model + CRP**  **OR (95%CI)** |
| --- | --- | --- |
| **Female gender** | 0.29 (0.16–0.51) | 0.29 (0.16–0.53) |
| **Pain duration 24–48 hours** | 1.70 (0.69–4.22) | 1.45 (0.56–3.75) |
| **Pain duration > 48 hours** | 0.79 (0.41–1.53) | 0.58 (0.28–1.17) |
| **RLQ tenderness** | 2.69 (1.21–5.98) | 2.21 (0.97–5.02) |
| **Abnormal bowel sounds** | 1.38 (0.57–3.34) | 1.03 (0.36–2.90) |
| **Peritoneal irritation** | 4.68 (2.34–9.36) | 4.46 (2.16_9.24) |
| **Elevated temperature** | 3.57 (1.87–6.81) | 3.03 (0.97–4.25) |
| **Nausea/ vomiting** | 2.37 (0.90–6.27) | 2.13 (0.75–6.03) |
| **CRP** | – | 1.020 (1.013–1.028) |

Abbreviations: CI, confidence interval; CRP, C-reactive protein; OR, odds ratio; RLQ, right lower quadrant.
