# Supplementary material for: Added value of CRP to clinical features when assessing appendicitis in children
Source: Eur J Gen Pract. 2022 May 10;28(1):95–101. doi: 10.1080/13814788.2022.2067142 (PMC9103685; doi:10.1080/13814788.2022.2067142)
Supplement: Supplementary Table 3 [file IGEN_A_2067142_SM8592.docx]

**Supplementary Table 3.** Diagnostic accuracy of CRP for the diagnosis of appendicitis at cut-off level of 10 mg/L: subgroup analyses for age and gender and symptom duration

|  | **Sensitivity (95%CI)** | **Specificity**  **(95%CI)** | **LH+**  **(95%CI)** | **LH- (95%CI)** | **PPV**  **(95%CI)** | **NPV**  **(95%CI)** |
| --- | --- | --- | --- | --- | --- | --- |
| **Age** | | | | | | |
| *4–8 years*  *(N = 190)* | 0.83  (0.44–0.97) | 0.70  (0.63–0.76) | 2.74  (1.8–4.16) | 0.24  (0.04–1.44) | 0.08  (0.06–0.12) | 0.99  (0.96–1.00) |
| *9–12 years*  *(N = 303)* | 0.82  (0.62–0.93) | 0.85  (0.80–0.89) | 5.47  (3.89–7.70) | 0.21  (0.09–0.52) | 0.30  (23–38) | 0.98  (0.96–0.99) |
| *13–18 years*  *(N = 583)* | 0.91  (0.78–0.96) | 0.75  (0.71–0.79) | 3.63  (3.04–4.32) | 0.18  (0.05–0.32) | 0.22  (19–25) | 0.99  (0.98.6–1.00) |
| **Gender** | | | | | | |
| *Male*  *(n = 459)* | 0.89  (0.77–0.95) | 0.75  (0.70–0.79) | 3.51  (2.89–4.25) | 0.14  (0.06–0.32) | 0.29  (25–33) | 98.4%  (96.4–99.3) |
| *Female*  *(N = 617)* | 0.83  (0.63–0.93) | 0.79  (0.75–0.82) | 3.83  (3.01–4.89) | 0.22  (0.09–0.54) | 0.13  (10–16) | 99.2%  98–99.7) |
| **Symptom duration (***N* **= 823) *** | | | | | | |
| *< 24 hours (N = 229)* | 0.67  (0.41–0.87) | 0.77  (0.71–0.82) | 2.87  (1.91–4.32) | 0.43  (0.23–0.84) | 0.20  (14–27) | 96.4%  (93.3–98.1) |
| *24– 48 hours*  *(N = 78)* | 0.91  (0.59–1.00) | 0.73  (0.61–0.83) | 3.38  (2.19–5.240) | 0.12  (0.02–0.81) | 0.36  (26–46) | 98.0%  (88.3–99.7) |
| *> 48 hours*  *(N = 516)* | 1.0  (0.89–1.00) | 0.75  (0.71–0.79) | 4.07  (3.48–4.75) | 0 | 0.21  (19–24) | 100% |

*Children with missing symptom duration were excluded from the analysis

Abbreviations: CI, confidence interval; CRP, C-reactive protein; LH+, positive likelihood ratio; LH-, negative likelihood ratio; NPV, negative predictive value; PPV, positive predictive value.
