# Supplementary material for: Added value of CRP to clinical features when assessing appendicitis in children
Source: Eur J Gen Pract. 2022 May 10;28(1):95–101. doi: 10.1080/13814788.2022.2067142 (PMC9103685; doi:10.1080/13814788.2022.2067142)
Supplement: Supplementary Table 2 [file IGEN_A_2067142_SM8591.docx]

**Supplementary Table 2.** Comparison of patients with and without missing values

|  | No missing values  *N* = 265 | | Missing Values  *N* = 811 | | Univariate OR (95%CI) |
| --- | --- | --- | --- | --- | --- |
|  | *N* | Value | *N* | Value |  |
| Age, median (IQR) | 219 | 13 (5) | 857 | 13 (6) | 1.01 (0.97-1-05) |
| Female, *N* (%) | 219 | 121 (55) | 857 | 496 (58) | 1.11 (0.83-1.50 |
| *–Duration of pain 24–48 hrs* | 219 | 30 (14) | 604 | 48 (8) | 0.69 (0.40-1.18) |
| *–Duration of pain > 48 hrs* | 219 | 120 (55) | 604 | 393 (66) | 1.42 (1.00-2.02) |
| Tenderness RLQ, *N* (%) | 219 | 134 (61) | 480 | 281 (59) | 0.90 (0.65-1.24) |
| Abnormal Bowel Sounds, *N* (%) | 219 | 33 (15) | 359 | 36 (10) | 0.63 (0.38-1.04) |
| Peritoneal Irritation, *N* (%) | 219 | 69 (32) | 506 | 124 (25) | 0.71 (0.50-1.00) |
| Elevated temperature, *N* (%) | 219 | 63 (29) | 499 | 164 (33) | 1.21 (0.86-1.72) |
| Nausea/Vomiting, *N* (%) | 219 | 144 (66) | 356 | 240 (68) | 1.08 (0.76-1.54) |
| CRP, median (IQR) | 219 | 5 (12) | 811 | 5 (10) | 0.998 (0.994-1.003) |
| Appendicitis, *N* (%) | 219 | 26 (12) | 857 | 44 (5.1) | 0.40 (0.24–0.67) |

Abbreviations: CI, confidence interval; CRP, C-reactive protein; IQR, interquartile range; OR, Odds Ratio.
